# New Chemotherapeutic Approaches to Treatment of Mesenchymal Triple-Negative Breast Cancer-Sensitive and Resistant to Cisplatin: Assessment of Cellular Response by Vibrational Microspectroscopy

**DOI:** 10.1021/acs.analchem.5c02233

**Published:** 2025-07-04

**Authors:** Clara B. Martins, Ana L. M. Batista de Carvalho, Maria M. Félix, Martin Vojtek, Carmen Diniz, Luís A. E. Batista de Carvalho, Maria P. M. Marques

**Affiliations:** † Molecular Physical-Chemistry − LAQV/REQUIMTE, Department of Chemistry, 37829 University of Coimbra, 3004-535 Coimbra, Portugal; ‡ Department of Life Sciences, Faculty of Science and Technology, University of Coimbra, 3000-456 Coimbra, Portugal; § LAQV/REQUIMTE, Laboratory of Pharmacology, Department of Drug Sciences, Faculty of Pharmacy, 26706 University of Porto, 4050-313 Porto, Portugal

## Abstract

Triple-negative breast cancer (TNBC) is the most aggressive type of breast tumor with the worst prognosis. New chemotherapeutic agents against TNBC are essential, aiming at a higher efficacy regarding cell growth inhibition and decreased angiogenesis and invasiveness coupled to lower acquired resistance and deleterious side effects. In this study, Raman and Fourier Transform Infrared (FTIR) microspectroscopies were applied to assess the impact of a trinuclear palladium-spermidine complex on human healthy and mesenchymal TNBC cells, with the results being compared to the clinically used drug cisplatin. To understand the metabolic impact of the drugs at the molecular level and identify the main biomarkers, unsupervised multivariate analysis of the data (Principal Component Analysis and Hierarchical Cluster Analysis) was applied to the vibrational data. The results revealed that the new palladium (Pd) agent had a higher effect on the cellular lipids relative to the platinum (Pt) compound (cisplatin), while the latter showed a stronger impact on the proteins. Besides lipids, Pd-agent showed a higher impact in conformational changes from the B-DNA native conformation to either Z- or A-DNA. This suggests the occurrence of distinct pathways of cytotoxicity for these metal complexes. Also, when comparing cisplatin-sensitive to cisplatin-resistant cells, Pd-agent had a more significant impact on νOPO_backbone_ from DNA, δCH_2_ from lipids and ν_s_CC_ring_ from phenylalanine of cisplatin-sensitive cells, while in cisplatin-resistant cells, proteins were the most affected cell components. These results provided spectral features specific to malignancy that led to discrimination between drug-treated and untreated cells. This knowledge is essential for the rational design of improved drugs with a higher efficiency coupled to lower toxicity.

## Graphical abstract

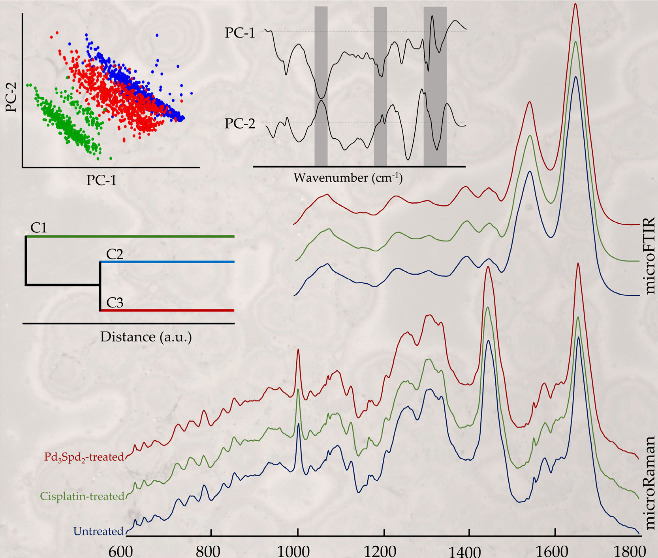

## Introduction

Cancer is the second leading cause of death globally, accounting for an estimated 670 000 deaths in 2022.^1^ Breast cancer is the most common cancer in women, with high mortality and increasing incidence (affecting one in every four).^2,3^ Despite all efforts regarding screening and treatment, this type of cancer remains a leading cause of death among women worldwide. In particular, triple-negative breast cancer (TNBC) is a subtype of breast tumor lacking hormone receptors expression, accounting for about 10–20% of the total number of breast cancer diagnosis, mainly in women younger than age 50.^4–6^ TNBC is a biologically aggressive tumor, characterized by the absence of estrogen receptor (ER), progesterone receptor (PR) and human epidermal growth factor receptor 2 (HER2), with high rates of metastasis and potential relapse, which, together with limited treatment options, leads to the poorest prognosis among breast subtypes.^7^ Currently, the luminal androgen receptor (LAR), basal-like immune-activated (BLIA), basal-like immune-suppressed (BLIS) and mesenchymal-like (M) subtype classifications of TNBC are widely accepted.^8,9^ Since the introduction of cisplatin (*cis*-Pt­(NH_3_)_2_Cl_2_) to the clinic in 1978,^10,11^ platinum complexes have been extensively studied as potential antineoplastic agents, leading to new platinum­(II) (Pt­(II)) anticancer drugs. Pt­(II) complex drugs, particularly those that target genomic DNA (e.g., cisplatin, carboplatin, and oxaliplatin), are widely used in the clinic to treat various types of cancer. The therapeutic effectivity of these agents results from the formation of Pt-DNA adducts through covalent binding to the bases, which causes damage to the DNA double helix, triggering cell death.^12–14^ Approximately 50% of cancer patients undergoing neoadjuvant chemotherapy receive a platinum drug either alone or in coadministration therapy. Nevertheless, the limited spectrum of antitumor activities, adverse side effects and repeated induction of resistance often lead to treatment failure.^15–17^ To overcome these drawbacks, substantial focus has been paid to the development of new metal-based agents with improved properties, selectivity and new action mechanisms.^18–24^ As potential Pt­(II) analogous, palladium­(II) (Pd­(II)) complexes have received attention due to similarities between these cations regarding both electronic configuration and coordination as well as due to their low toxicity compared to conventional chemotherapeutics.^21,22,25–32^ These complexes with more than one metal center have the ability to bind to the DNA’s helix in more than one site and through long-range interstrand adducts, which prompts more severe and irreparable damage compared to conventional mononuclear Pt­(II) drugs. In addition to DNA, other targets of Pd complexes have been described, such as proteins,^33^ oxidative species^34,35^ or intracellular water.^36,37^ Biogenic polyamines, namely spermidine (H_2_N­(CH_2_)_4_NH­(CH_2_)_3_NH_2_, Spd) have been identified as suitable polydentate ligands for both Pt^2+^ and Pd^2+^ ions, allowing the formation of stable chelates, such as Pt­(II) or Pd­(II) chelates with spermidine^22,29,31,32,38^ (Pd_3_Spd_2_, Figure 1).

**Figure 1:**
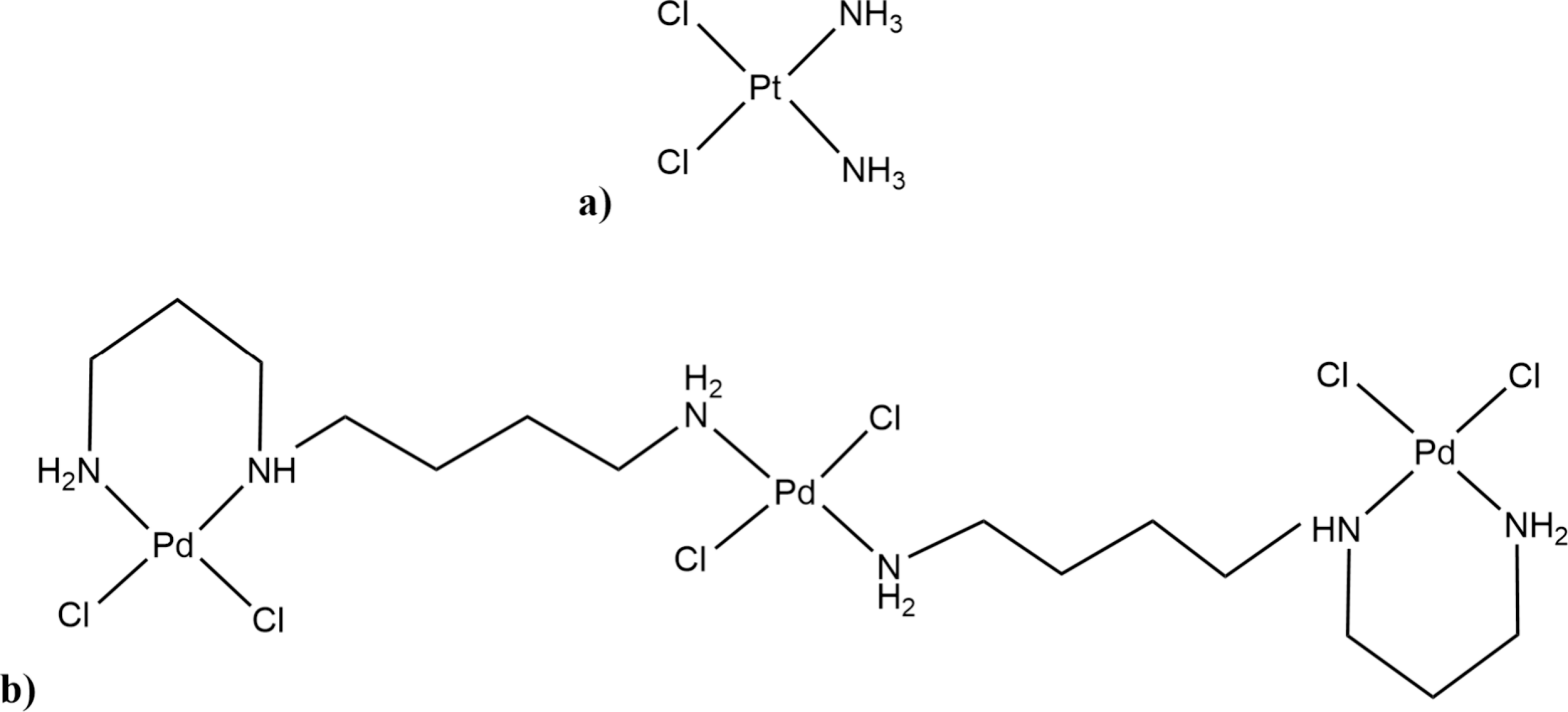
Structure of a) Pd­(II)-based agents: Pd­(II) trinuclear chelate with spermidine (Pd_3_Spd_2_) and b) conventional mononuclear Pt­(II) drug cisplatin.

Pd­(II) chelate with spermidine (Pd_3_Spd_2_) have been reported to yield differentiated anticancer activity toward triple-negative breast cancer.^32^ This result evidence the promising therapeutic potential of Pd­(II) chelates with biogenic polyamines and fosters further research on other similar complexes.

Raman and Fourier transform infrared (FTIR) spectroscopies are complementary techniques with high specificity and spatial resolution. They are promising tools in medicinal chemistry since they are noninvasive, reproducible, and cost-effective. Coupled to optical microscopy, they allow us to analyze heterogeneous biological samples such as tissues and cells (both fixed and live), with a view to obtain biochemical information for building chemical images,^39–44^ allowing us to determine the metabolic impact and mode of action of chemotherapeutic agents, as well as the cellular response to treatment.^22,31,38,45^ The identification of spectral biomarkers of drug action is paramount for understanding the impact of new chemotherapeutic agents in cells, enabling the development of improved anticancer drugs.

The present work aimed to assess the metabolic impact of the trinuclear Pd­(II) complex with spermidine as the biogenic polyamine, synthesized and fully characterized by the authors, Pd_3_Spd_2_Cl_6_ (hereafter denominated Pd_2_Spd_3_), as well as cisplatin on (i) mesenchymal TNBC cisplatin-sensitive cells (MDA-MB-231), (ii) mesenchymal TNBC cisplatin-resistant cells (MDA-MB-231/R) and (iii) healthy human breast cells (MCF-12A). Vibrational microspectroscopy, namely microRaman and microFTIR, were used in order to assess the drugs’ bioavailability, as well as cellular response to treatment drugs. The data obtained within this study, coupled to cytological assays (for evaluation of growth-inhibition, antiproliferative effect) previously carried out by the authors^32^ will pave the way for future studies on the rational design of novel drugs displaying a higher efficiency, lower toxicity, decreased acquired resistance and possible oral administration.

## Experimental Section

The list of all chemicals as well as the details of the synthesis of Pd_3_Spd_2_, cell culture procedure, sample preparation for vibrational microspectroscopy measurements, data processing, and statistical analysis are described in the Supporting Information.

### Cell Culture

Resistant mesenchymal TNBC cell lines (MDA-MB-231/R) and cisplatin-sensitive TNBC cell lines (MDA-MB-231) were investigated in this work and compared with human healthy breast cells (MCF-12A). All cell lines were cultured in monolayers, at 37 °C, in a humidified atmosphere of 5% CO_2_. The tested agents were added to the cells when they were in the respective exponential phase of growth.

### Sample Preparation for Vibrational Microspectroscopy Measurements

A cell concentration of 1.5 × 10^4^ cells/cm^2^ was seeded onto optical substrates suitable for either Raman or FTIR acquisition. After an incubation period of 24 h, the cells were treated with either Pd_3_Spd_2_ or cisplatin at the respective IC_50_ values (50% cell growth inhibition value) previously determined by the authors,^32^ respectively, for Pd_3_Spd_2_ and cisplatin: 4.65 μM and 1 μM for MDA-MB-231, 10.57 μM and 32.4 μM for MDA-MB-231/R, 53 μM and 1 μM for MCF-12A. Upon 48 h of drug exposure, the cells were fixed with 4% formalin. All samples were prepared in triplicate in three independent assays.

### FTIR Microspectroscopy

The microFTIR spectra were acquired in the mid-IR range (400–4000 cm^–1^), using a Bruker Hyperion 2000 microscope with a liquid nitrogen-cooled mercury–cadmium–telluride (MCT) detector, in transmission mode using a 15× Cassegrain both condenser and objective, coupled to a Bruker Optics Vertex 70 spectrometer, both purged by CO_2_-free dry air. Each spectrum was the sum of 128 scans (for both the sample and background) at a 4 cm^–1^ resolution. The background was measured every 10 spectra on a clear area of the CaF_2_ disk. 150 spectra were collected per sample: 1 spectrum for each cell, from random regions (ca. 150 cells were probed per sample).

### Raman Microspectroscopy

The microRaman spectra were recorded in an Oxford Instruments WITec (Ulm, Germany) confocal Raman microscope system alpha 300R coupled to an ultrahigh-throughput spectrometer (UHTS) 300 VIS-NIR, using a 532 nm diode-pumped solid-state laser. The measurements were acquired using a 100×/0.8 Zeiss Epiplan objective, with 3 accumulations of 15 s per spectrum. 150 spectra were collected per sample: 1 spectrum for each cell, from random region (ca. 150 cells were probed per sample).

### Data Processing and Statistical Analysis

The microFTIR data spectra were preprocessed using the OPUS 8.1 software: atmospheric compensation baseline correction. The spectra were quality checked (to remove data with high levels of scattering and fixed-pattern noise) based on the amide I band intensity, with spectra with absorbances between 0.1 and 1 being retained. Mie scattering was corrected using an Extended Multiplicative Signal Correction (EMSC) function with 20 iterations.^46^ The data were cropped into two spectral ranges: the fingerprint region (1000–1800 cm^–1^) and the high-wavenumber interval (2700–3650 cm^–1^).

The microRaman spectra were preprocessed using Project FIVE software (WITec). The spectra were cropped to the fingerprint (600–1800 cm^–1^) and high-wavenumber (2600–3700 cm^–1^) regions and the signal background. The cosmic-ray spikes were identified and removed. A principal-component (PC)-based noise reduction algorithm was applied by retaining a selected number of principal components (20 PC’s) and then recombing the data set. The spectra were vector normalized and mean-centered before Principal Component Analysis (PCA). Multivariate data analysis was applied, unsupervised machine learning techniques such as PCA and Hierarchical Cluster Analysis (HCA), using Matlab R2024b (MathWorks) and Quasar Spectroscopy 1.10.1 software. The order of the PC’s denotes their importance, PC-1 corresponding to the highest amount of variation. In HCA, one or more spectral regions are grouped according to their degree of similarity, and a dendrogram of the mean fingerprint FTIR and Raman spectra was performed. Manhattan metric distances were calculated between the spectra of cells, and then the Ward method was applied. All spectra were grouped in three clusters with the highest heterogeneity.^47,48^ A confusion table was generated, displaying the number of predicted spectra vs true classes, with a view to distinguish untreated from Pd_3_Spd_2_-treated cells. Specificity was defined as the fraction of correctly predicted negatives from the total number of true negatives. Sensitivity was calculated as the fraction of correctly predicted positives from the total number of true positives. This analysis was performed using MATLAB R2024b (MathWorks).

## Results and Discussion

According to the promising results previously obtained by some of the authors^32^ from the biological assays for Pd_3_Spd_2_ and cisplatin for TNBC MDA-MB-231 and MDA-MB-231/R cell lines and for MCF-12A healthy breast cell line, the IC_50_ concentrations at 48 h were chosen (4.65 μM and 1 μM for cisplatin-sensitive TNBC cells, 10.57 μM and 32.4 μM for cisplatin-resistance TNBC cells and 53 μM and 1 μM, respectively) for the current study.

Figures 2 and 3 depict the average spectra obtained for each experimental condition, containing information on the cellular biochemical profile, by microRaman and microFTIR. The Raman and FTIR spectra were randomly acquired as single point spectra (150 spectra per sample) from disks comprising cells in different phases of the cell cycle, exhibiting a high degree of heterogeneity. Attending to that, a large amount of data was required to represent the sample and minimize the effect of the cell cycle profile, enabling an effective comparison between treated and untreated cells This approach allows multivariate analysis techniques to consider all available vibrational information, increasing the likelihood of detecting biochemical changes that may not be evident when analyzing only specific bands.

**Figure 2:**
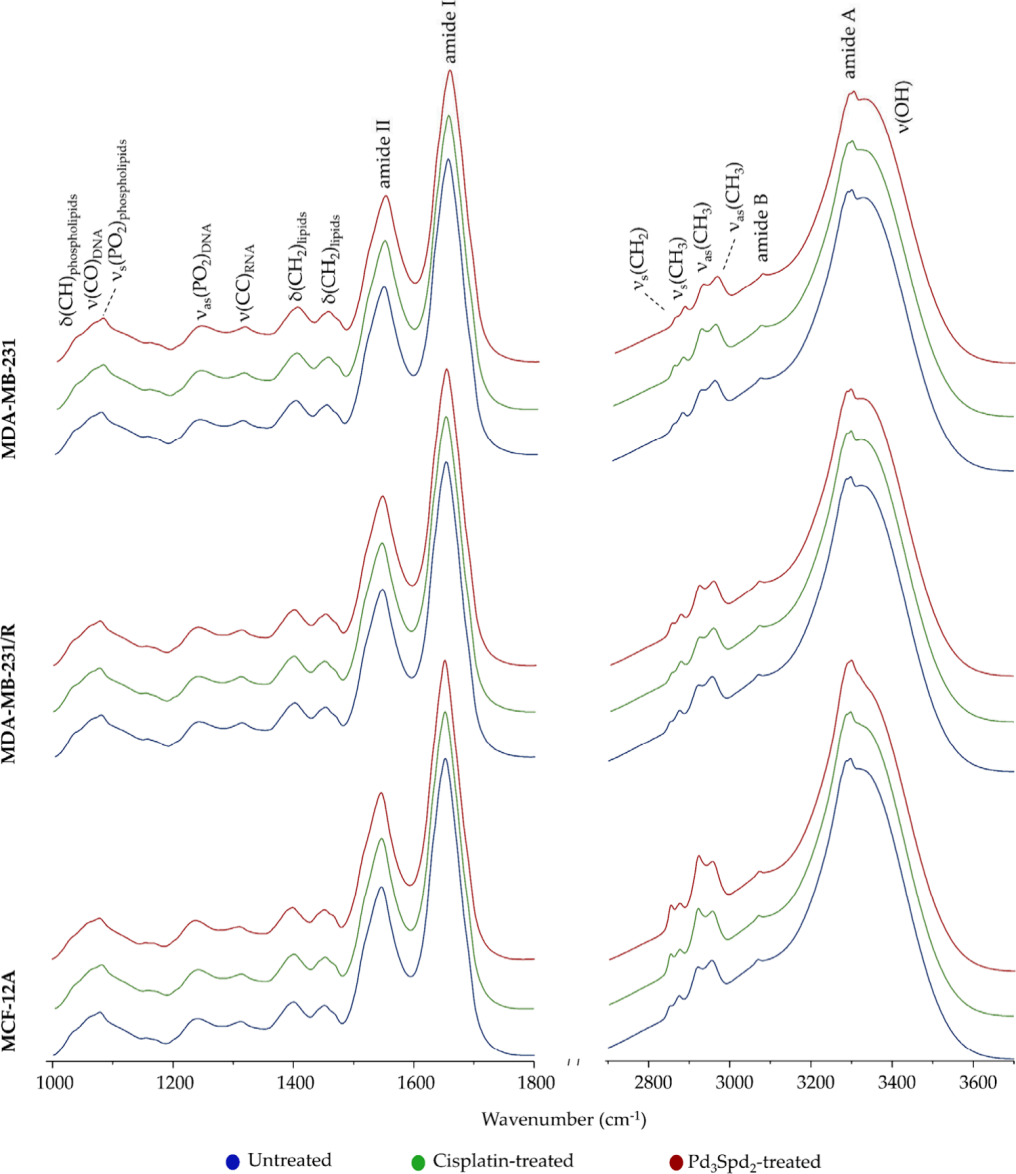
Mean FTIR spectra (1000–1800 cm^–1^ and 2700–3650 cm^–1^) for healthy human breast cell line MCF-12A and human breast cancer cell lines MDA-MB-231 and MDA-MB-231/R. (δ - deformation; ν - stretching; s - symmetric; as - antisymmetric).

**Figure 3:**
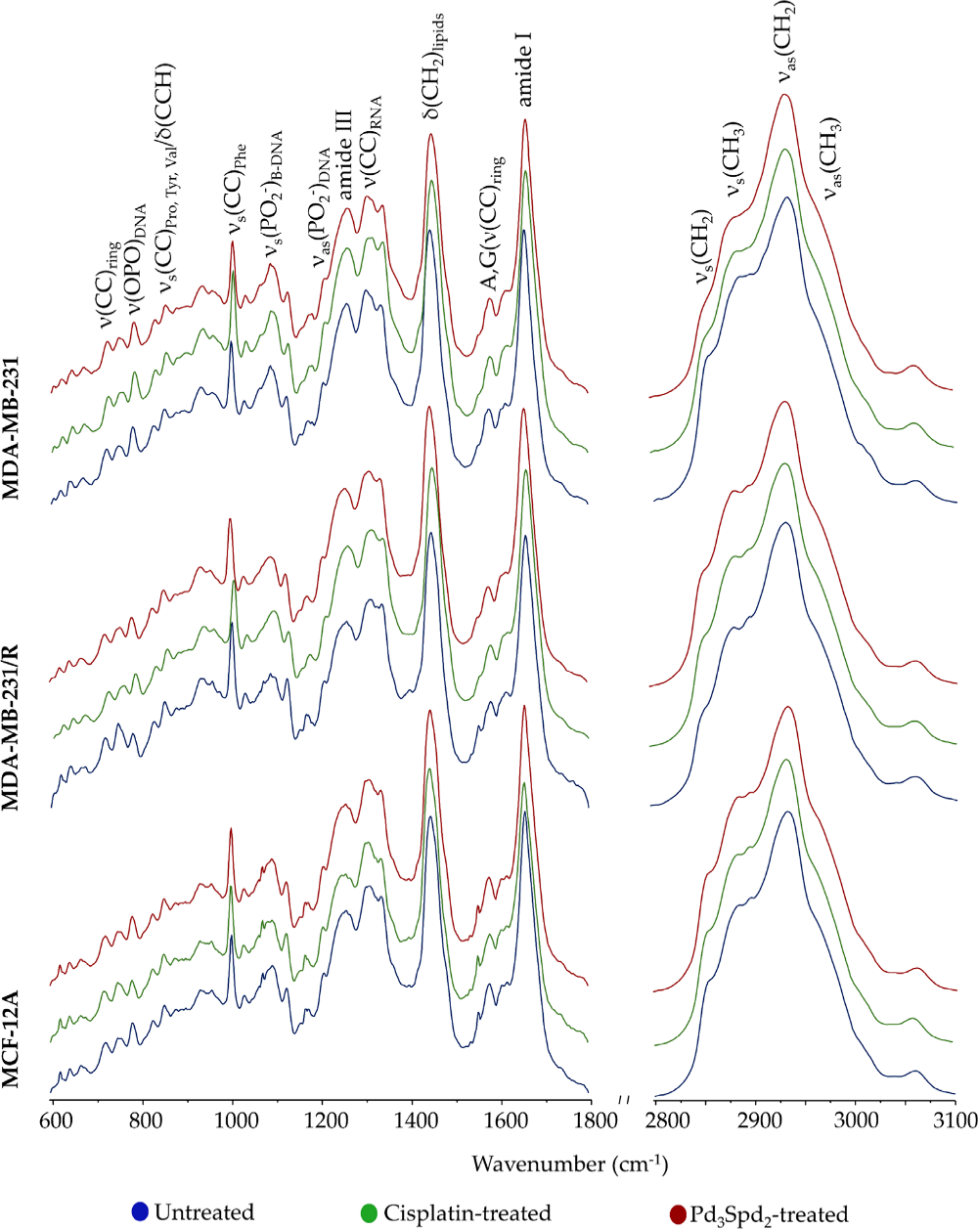
Mean Raman spectra (600–1800 cm^–1^ and 2800–3050 cm^–1^) for healthy human breast cell line MCF-12A and human breast cancer cell lines MDA-MB-231 and MDA-MB-231/R. (δ - deformation; ν - stretching; s - symmetric; as - antisymmetric. Phe - phenylalanine; Pro - proline; Tyr - tyrosine; Val - valine).

In this study, the metabolic impact of the Pd­(II) compound on mesenchymal TNBC cell lines and on healthy breast cell line was compared with cisplatin, since its binding to DNA and its mechanism of action are well-known: upon intracellular hydrolysis of chloride leaving ligands, the unstable diaquo complex is formed and covalent binding to the nucleophilic sites of DNA occurs, primarily the N7 atoms of purine bases, forming 1,2- and 1,3-intrastrand cross-links and, less frequently, interstrand adducts. This triggers cell cycle arrest and apoptotic death.^49,50^

Since the Raman and FTIR spectroscopic results have high variability, it was necessary to apply multivariate analysis techniques to interpret spectral information, unveiling the chemical differences between the control and drug-treated cells for the healthy and TNBC cell lines.

In general, good discrimination in PCA and separation in HCA were attained for both microspectroscopies, with a slight overlap between drug exposure and untreated cells observed in the FTIR data for MDA-MB-231 and MDA-MB-231/R cells and in the Raman data for the healthy cell line.

Table S1 (Supporting Information) comprises the Raman and FTIR assignments proposed for the cell lines under study, illustrating their biochemical profile. Several specific biomarkers such as DNA and protein conformational rearrangements were identified due to the clear spectral changes triggered by the Pd_3_Spd_2_ complex.

### MDA-MB-231 Cell Line

Overall, the spectral data allowed differentiation of the untreated MDA-MB-231 cell line from the MDA-MB-231 cell line treated with Pd_3_Spd_2_ and treated with cisplatin. The chemical differences were clearly unveiled upon PCA analysis of the spectroscopic results (Figure 4).

**Figure 4:**
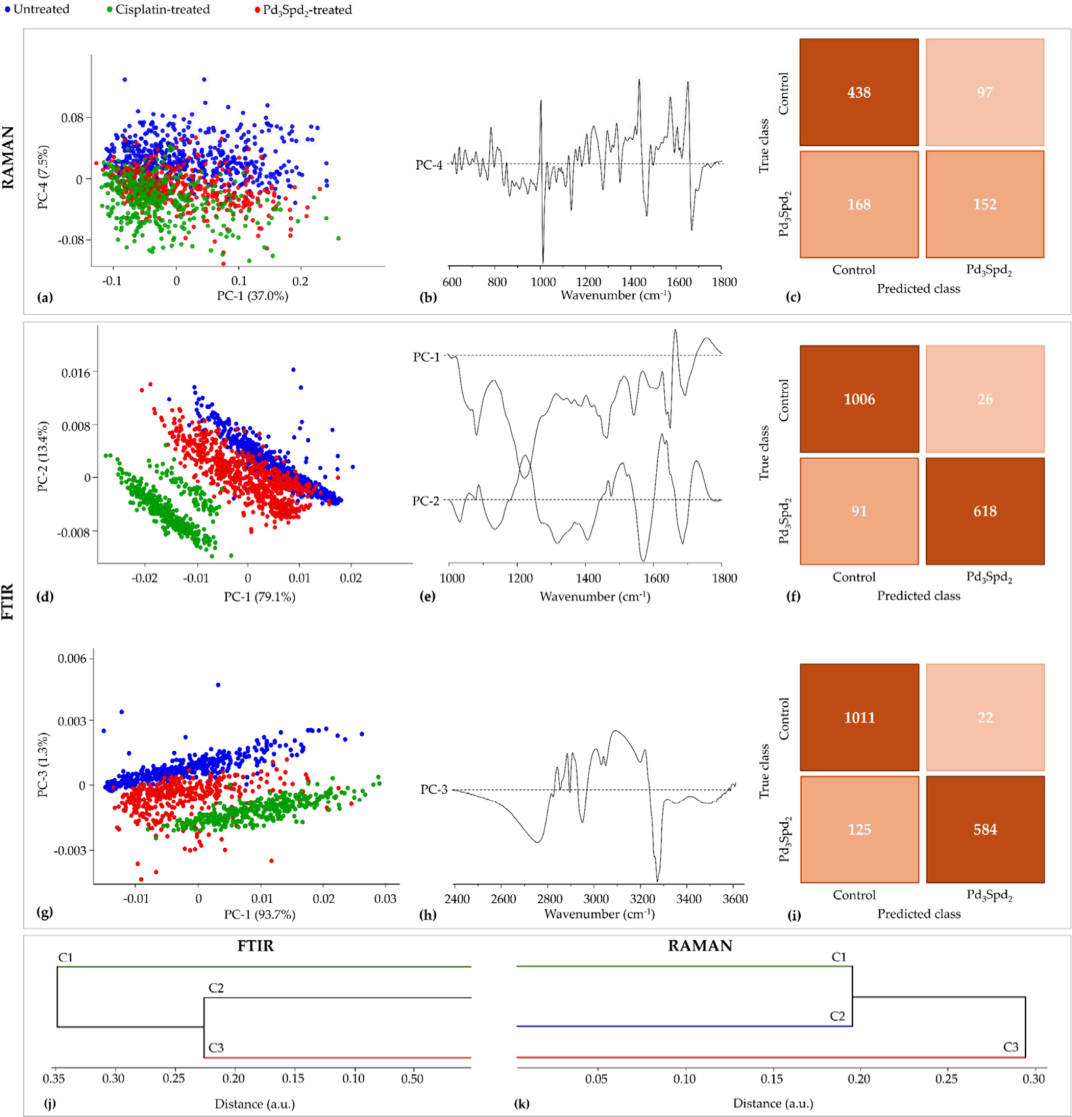
PCA and HCA of Raman and FTIR data for cisplatin-sensitive cell line MDA-MB-231 treated with cisplatin and Pd_3_Spd_2_ vs untreated cells. (a,b) Score and loading plots of the Raman fingerprint region. (c) Confusion table of the FTIR classification model tested on Pd_3_Spd_2_ data. (d,e) Score and loading plots of the FTIR fingerprint region. (g,h) Score and loading plots of the FTIR high-wavenumber region. (f,i) Confusion tables of the FTIR fingerprint (f) and high-wavenumber (i) classification models tested on Pd_3_Spd_2_ data. (j,k) HCA dendrogram of the mean FTIR (j) and Raman (k) spectra.

Regarding the Raman data of Pd_3_Spd_2_- and cisplatin-treated MDA-MB-231 cells in comparison with data for the untreated control (Figure 4a,b), the main differences were found in the fingerprint region along PC-4, explaining 7.5% of the total data variance, with only a small overlapping of drugs treated cells.

Figure 5 shows the pairwise analysis of the Raman data (cisplatin-treated cells vs untreated cells (Figure 5a,b) and Pd_3_Spd_2_-treated vs untreated cells (Figure 5c,d) for better understanding. The chosen PCs have a low percentage of variance; however, the sum of all the initial PCs (PC1 to PC4) still explains a substantial part of the variance, which was proven by separating the conditions in the study.

**Figure 5:**
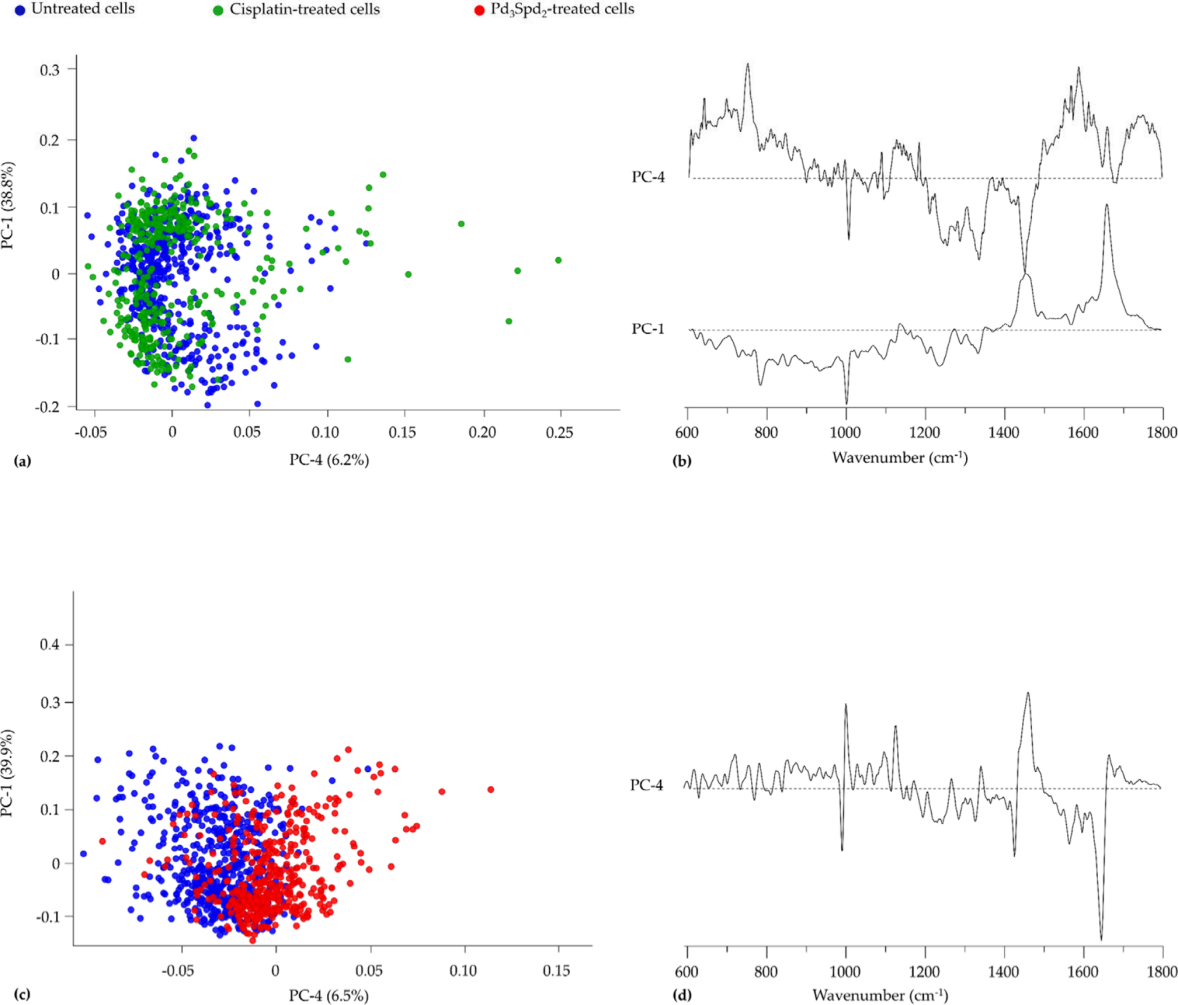
PCA of Raman data for cisplatin-sensitive cell line MDA-MB-231 treated with (a,b) cisplatin vs untreated cells and (c,d) Pd_3_Spd_2_ vs untreated cells.

Each principal component corresponded to the loading plots, which provided information on the main differences between the three experimental conditions, mainly along PC-4 (Figure 4a): treated cells showed a higher contribution of vibrational modes at 1034 cm^–1^ (νCC_lipids_, δCH phospholipids, (νCC, νCO, νC–OH)_carbohydrates_) and at 1272 cm^–1^ (amide III/α-helix, (δCH_2_, ωCH_2_, tCH_2_)_lipids_) (Table S1). Changes in the bands associated with the DNA phosphate groups such as νOPO_backbone_ at 785 cm^–1^ are characteristics of B-DNA, and ν_s_PO_2_^–^ at 1092 cm^–1^ and ν_as_PO_2_^–^ at 1238 cm^–1^ are informative about a drug-prompted distortion of the double helix. Treated MDA-MB-231 cell line had a higher contribution from the νOPO_backbone_ of Z-DNA at 863 cm^–1^. The transition from DNA’s native B- to Z-DNA conformation suggests that the drugs effectively bind to the double helix and induce its disruption, through formation of cross-links that interfere with the base-stacking and/or base-pairing, thus preventing the reversal of this damage by the cell’s repair mechanisms.^50^ These results are in line with studies carried out previously by the authors’ lab.^26,30,31^ In addition, the variations in the nitrogenous bases—νCC_ring_ adenine, guanine, cytosine, and thymine at 1373 cm^–1^, 1338 cm^–1^ and 1175 cm^–1^—showed the presence of drug–DNA cross-links, predominantly interstrand, leads to local unwinding of the native helix. In light of these results, and as has been pointed out by the authors,^26,30,31,51^ the main target of the palladium complexes with biogenic polyamines is the DNA double helix. Regarding proteins, the main discrimination observed was in the ν_s_CC_ring_ of phenylalanine at 1005 cm^–1^ and in the δCH and νO–CH_3_ bands at 1034 cm^–1^. The integrity of untreated cells is proven by DNA signals: B-DNA/dG νCC_ring_, Met νCS, phosphate esters ν_s_OPO at 698 cm^–1^, B-DNA/T νCC_ring_ at 746 cm^–1^, C,T,U νCC_ring_ at 777 cm^–1^ and G νCC_ring_ at 1338 cm^–1^. Lipids are known to be an excellent source of information on cancer growth and MDA-MB-231 cells are rich in lipids, such as glycerophospholipids, known to be increased in cancer cells.^52,53^ The drug effect on these cells was reflected in an increase in the intensity of the δCH_2_ Raman band at 1432 cm^–1^.

The FTIR data revealed a much more significant differentiation between untreated and treated cells. A clear separation was observed in the spectral fingerprint region for the three conditions evidenced along PC-1 with 79.1% of total variance (Figure 4d,e) followed by PC-2 (13.4%). Concerning protein content, especially in ν­(CC) and ν­(CN) vibrational modes at 1080 cm^–1^, amide II (δCN-H/νCN) at 1545 cm^–1^, amide I at 1650–1660 cm^–1^ and νC=O of the amino acids side chain at 1690 cm^–1^ showed a higher damage in the presence of cisplatin. These vibrational modes, assigned to cellular proteins, suggest that the drug acts either by binding to cytoplasmic proteins or via proteolysis during apoptosis.^26^ On the other hand, Pd_3_Spd_2_ has a significant impact on δCH_2_ from lipids at 1450 cm^–1^ and on phospholipids (νC=O_ester_) at 1724 cm^–1^. The combination PC-1 (93.7%) vs PC-3 (1.3%) enables a clear separation of untreated from cisplatin-treated and Pd_3_Spd_2_-treated cells in the spectral high-wavenumber region (Figure 4g,h). The main differentiating signals from the untreated cells are centered at 2852 and 2915 cm^–1^. At the proteins level, a higher contribution of ν_s_CH_2_ at 2852 cm^–1^ and ν_as_CH_2_ at 2900 – 2935 cm^–1^ was verified for cisplatin-treated cells, and less in Pd_3_Spd_2_-treated and untreated cells (Table S1).

According to the dendrograms obtained by HCA (Figure 4j,k), the three conditions can be completely separated into different clusters in both techniques. In the FTIR, the untreated cells and Pd_3_Spd_2_-treated cells have greater similarity, which is demonstrated in the PCA through the slight overlaps of both conditions. The higher level of similarity between untreated and cisplatin-treated cells was observed in the Raman.

Figure 4c,f,i shows the cross-validation confusion tables for the Raman and FTIR classification model, concerning the comparison between the cisplatin-sensitive MDA-MB-231 cell line treated and untreated with Pd_3_Spd_2_. The confusion table of the Raman data demonstrated that among 320 spectra from the Pd_3_Spd_2_ treatment group, 168 (53%) were misclassified as control group, signifying that 152 spectra were correctly classified (Figure 4c). This reflects the incomplete untreated and treated cell separation already evidenced in the corresponding PCA score (Figure 4a). The confusion table of the FTIR fingerprint region indicates that among 1032 spectra from the control, only 26 (2.5%) were misclassified as Pd_3_Spd_2_-treated cells (Figure 4f). On the other hand, 618 out 709 spectra were accurately classified as belonging to the Pd_3_Spd_2_-treated cells. Regarding the confusion table of FTIR high-wavenumber region, 1011 out of 1033 were correctly classified as control and only 18% of Pd_3_Spd_2_-treated cells spectra were misclassified as belonging to the control group (Figure 4i).

### MDA-MB-231/R Cell Line

Regarding the MDA-MB-231/R cell line, the Raman data yielded a much less significant differentiation between the treatments when compared to infrared data. An overlap of Pd_3_Spd_2_-treated cells and cisplatin-treated cells was registered in the Raman fingerprint region. However, a separation between untreated and treated cells was observed along PC-1 (34.5%) in combination with PC-3 (16.5%) for the fingerprint interval (Figure 6a,b).

**Figure 6:**
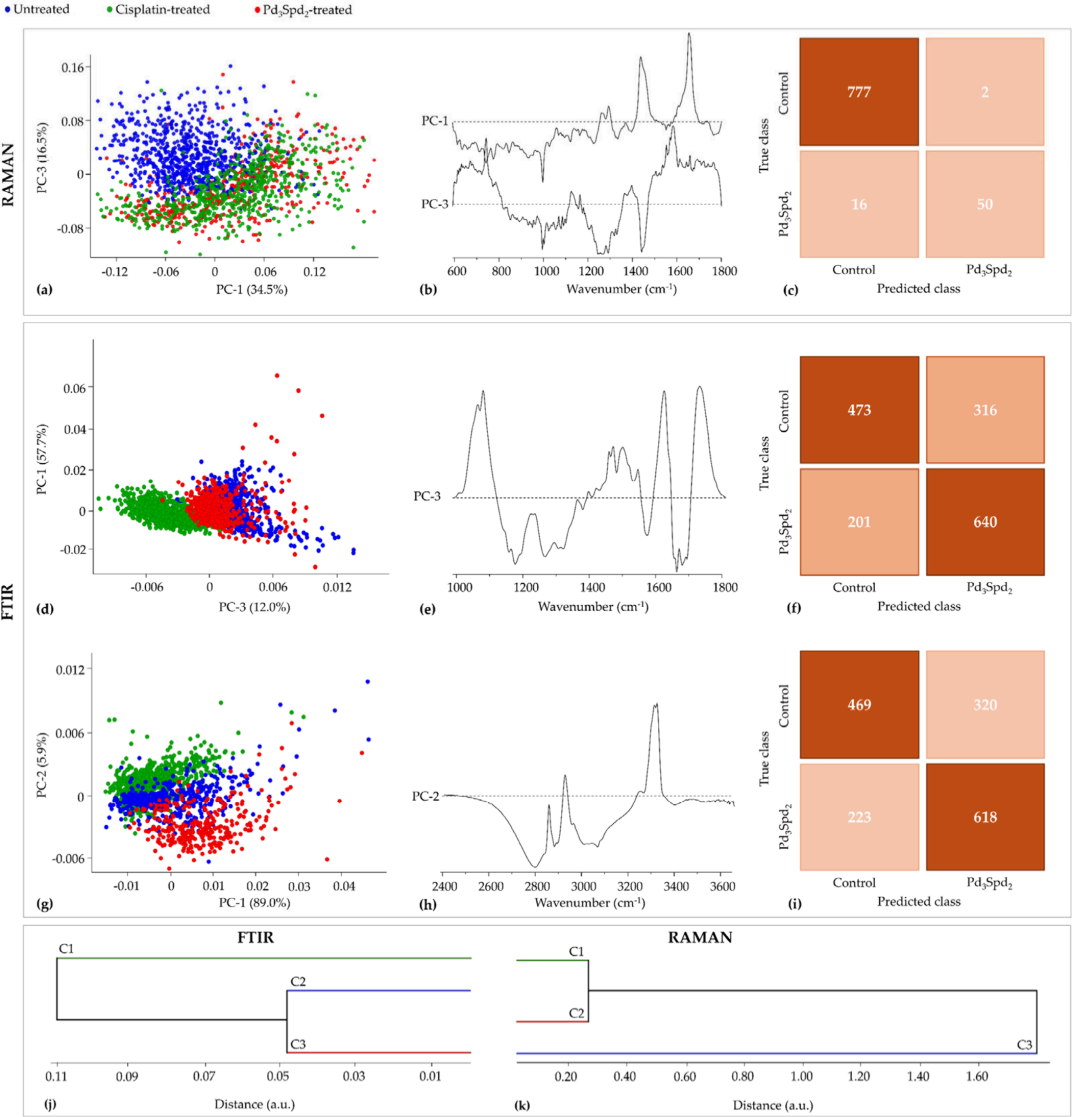
PCA and HCA of Raman and FTIR data for cisplatin-resistant cell line MDA-MB-231 treated with cisplatin and Pd_3_Spd_2_ vs untreated cells. (a,b) Score and loading plots of the Raman fingerprint region. (c) Confusion table of the FTIR classification model tested on Pd_3_Spd_2_ data. (d,e) Score and loading plots of the FTIR fingerprint region. (g,h) Score and loading plots of the FTIR high-wavenumber region. (f,i) Confusion tables of the FTIR fingerprint (f) and high-wavenumber (i) classification models tested on Pd_3_Spd_2_ data. (j,k) HCA dendrogram of the mean FTIR (j) and Raman (k) spectra.

Figure 7 clearly shows a distinct separation between the Raman data processing conditions. This separation is mainly due to a stronger contribution from vibrational modes: ν_s_CC_ring_ from phenylalanine at 1005 cm^–1^, νCO from RNA/ribose, νCN from proteins, νCC from acyl (*trans* conformation) lipids, νCO and νCC from carbohydrates at 1128 cm^–1^, νCC_ring_ from RNA/A,C at 1302 cm^–1^, amide I (νC=O/α-helix) at 1659 cm^–1^ and νC=O from amino acid side chain) at 1690 cm^–1^ for treated cells (Table S1). Lipids revealed a higher sensitivity to drug administration, especially in δCH_2_ at 1442 cm^–1^ and in νC=O_ester_ from phospholipids at 1732 cm^–1^. Treated MDA-MB-231/R cells showed a higher contribution from amide II (δCN-H/νCN) at 1545 cm^–1^, a result that was also found for MDA-MB-231 cells, with cisplatin having the greatest impact.

**Figure 7:**
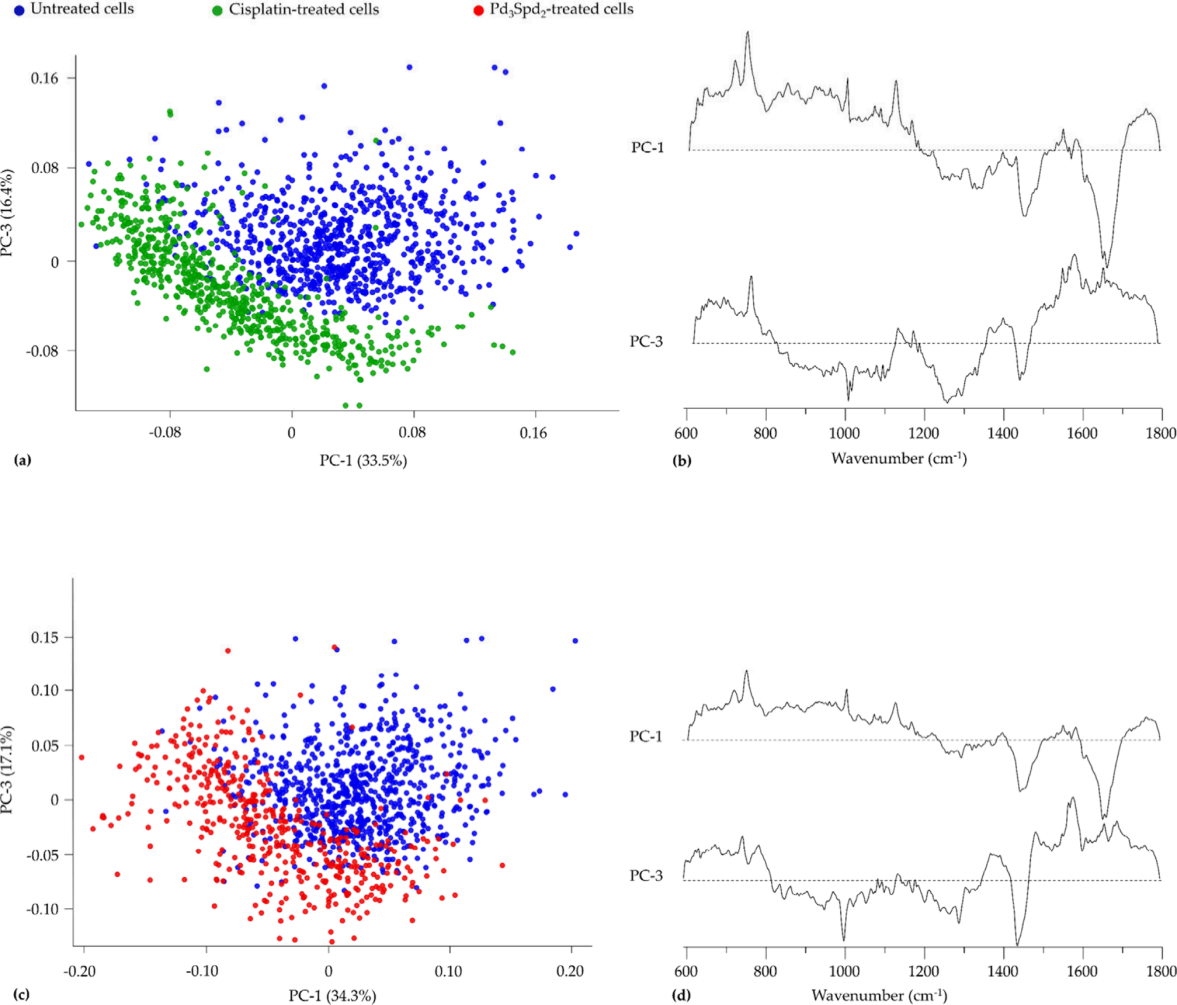
PCA of Raman data for cisplatin-resistant cell line MDA-MB-231 treated with (a,b) cisplatin vs untreated cells and (c,d) Pd_3_Spd_2_ vs untreated cells.

Concerning the FTIR data of the fingerprint region, there was a good separation of the three conditions under study, with a slight overlap between the administration of Pd_3_Spd_2_ and the untreated cells. PC-1 represented the main discrimination explaining 57.7% of total variance, followed by PC-3 (12.0%) (Figure 6d,e): Cisplatin displayed a higher contribution of DNA nucleobases bands (νCC_ring_ from adenine, guanine, cytosine, and thymine bases) at 1373 cm^–1^, δCH_2_ from A-DNA 1420 cm^–1^ (Raman) and νC=O from A-DNA at 1714 cm^–1^ (IR) (Table S1). Besides DNA, proteins were also affected by cisplatin, more precisely the vibrational modes δCH, νO–CH_3_ from phenylalanine at 1034 cm^–1^, νCC and νCN at 1080 cm^–1^, νCN at 1128 cm^–1^ and the νC=O from the amino acids side chain at 1690 cm^–1^. Changes in phospholipids (δCH, νCC) at 1034 cm^–1^, (ν_s_PO_2_^–^) at 1080 cm^–1^, and (νC=O_ester_) at 1724 cm^–1^ may be indicative of a drug interaction with the cellular membrane. Also, vibrational modes like the νCC from acyl (*trans* conformation) lipids, νCO and νCC from carbohydrates at 1128 cm^–1^, δCH_3_ from glycoproteins, δCH_3_ from lipids/acyl chains and δCH_2_ from saccharides at 1373 cm^–1^ showed higher contribution in treated cells. Pd_3_Spd_2_-treated cells and the control group have higher contributions from DNA bands (νCC_ring_ from cytosine, guanine, and thymine bases) at 1175 cm^–1^ and at 1650–1660 cm^–1^ (δNH). Concerning proteins, MDA-MB-231/R treated with Pd_3_Spd_2_ and cisplatin showed an impact on δCH from tyrosine and phenylalanine at 1175 cm^–1^ and amide I (νC=O/α-helix) at 1650–1660 cm^–1^ (Table S1). In the high-wavenumber region, the main discrimination was observed along PC-1 followed by PC-2 (explaining 89.0% and 5.9% of the total data variance, respectively) (Figure 6g,h). As expected, and considering the changes in the fingerprint region, separation was evident based on the vibrational modes (ν_s_CH_2_ from proteins, ν_s_CH and *v*_s_CH_2_ from lipids) at 2850 cm^–1^ and (ν_as_CH_2_ from proteins and ν_as_CH_2_ from lipids) at 2900–2935 cm^–1^. The amide A modes at 3250–3295 cm^–1^ were also found to undergo cisplatin-induced changes, which supports the impact of this drug on proteins.

The HCA in cisplatin-resistant cells for the FTIR data (Figure 6j) presented a higher similarity between the untreated cells and Pd_3_Spd_2_-treated cells, and for Raman (Figure 6k) a higher similarity between the cells treated with the drugs. The same can be concluded by the interpretation of both PCA scores.

The accuracy of the microspectroscopy methods applied for analysis of the metabolic impact of Pd_3_Spd_2_ on cisplatin-resistant cell line MDA-MB-231 is demonstrated by the confusion tables (Figure 6c,f,i). The confusion table of the Raman spectra demonstrated that among 779 spectra from the control group, only 2 were misclassified as Pd_3_Spd_2_-treated cells, meaning that 777 spectra were correctly classified as control (Figure 6c). The FTIR fingerprint region confusion table indicates that among 789 spectra from the control, 316 (40%) were misclassified as Pd_3_Spd_2_-treated cells (Figure 6f). On the other hand, 640 out of 841 spectra were accurately classified as belonging to the Pd_3_Spd_2_-treated cells. With regard to the FTIR high-wavenumber region confusion table, 469 out of 789 were correctly classified as control, 27% of Pd_3_Spd_2_-treated cells spectra was wrongly classified as belonging to the control group, and 73% of total spectra was correctly classified as Pd_3_Spd_2_-treated cells (Figure 6i).

### MCF-12A Cell Line

With regard to the Raman data (Figure 8a,b), separation between MCF-12A untreated vs Pd_3_Spd_2_-treated cells and cisplatin-treated cells was obtained along PC-2 followed by PC-4 (28.2% and 6.1% of total variance, respectively), where the untreated healthy cells and cisplatin-treated cells were clearly separated, but Pd_3_Spd_2_-treated cells overlap both conditions.

**Figure 8:**
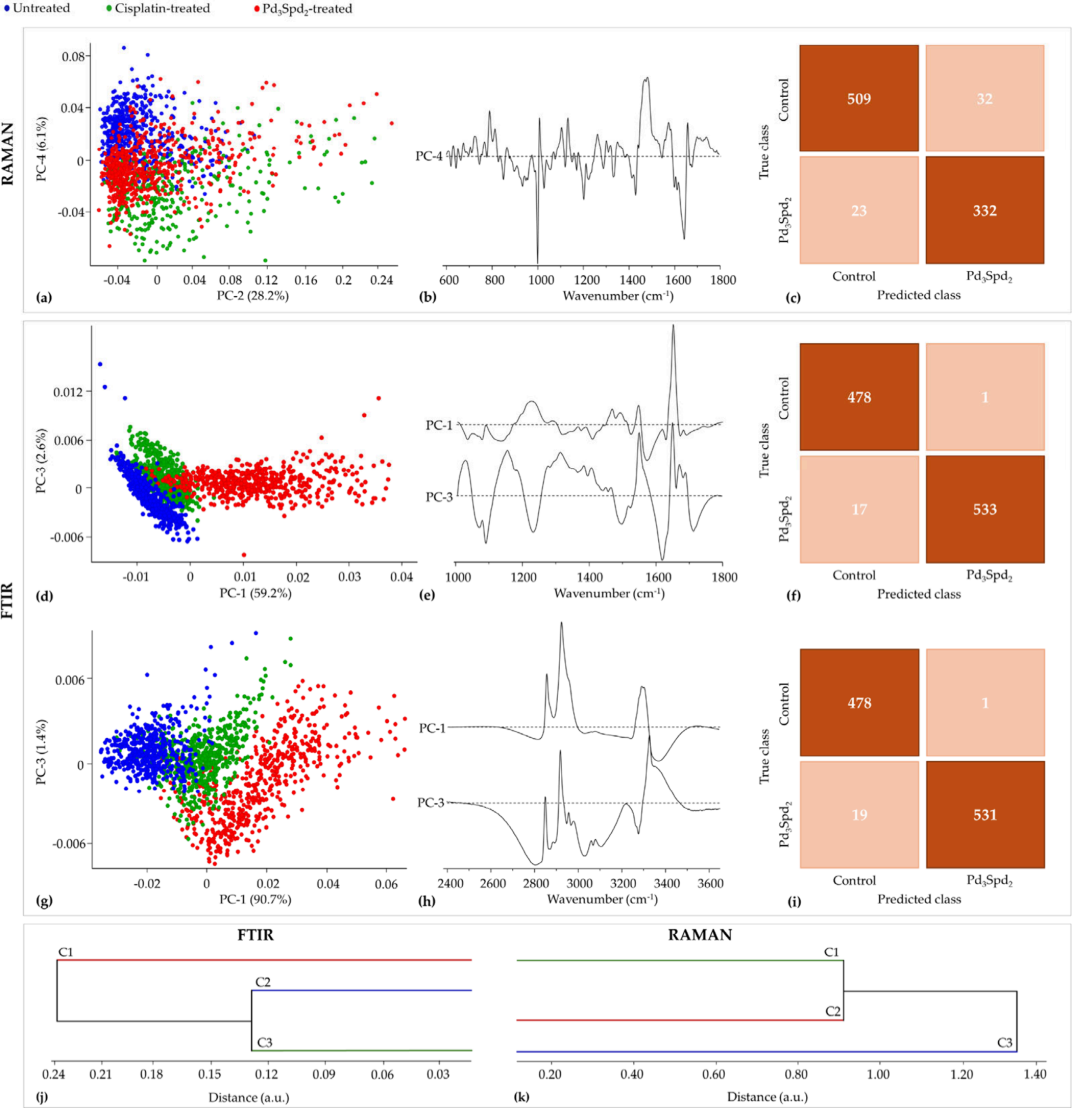
PCA and HCA of Raman and FTIR data for healthy breast cell line MCF-12A treated with cisplatin and Pd_3_Spd_2_ vs untreated cells. (a,b) Score and loading plots of the Raman fingerprint region. (c) Confusion table of the FTIR classification model tested on Pd_3_Spd_2_ data, (d,e) Score and loading plots of the FTIR fingerprint region. (g,h) Score and loading plots of the FTIR high-wavenumber region. (f,i) Confusion tables of the FTIR fingerprint (f) and high-wavenumber (i) classification models tested on Pd_3_Spd_2_ data. (j,k) HCA dendrogram of the mean FTIR (j) and Raman (k) spectra.

A pairwise analysis of the Raman data was performed to clarify the differences between the treatments (Figure 9). As mentioned above, the sum of all the initial PCs (PC1 to PC4) still explains 93% of total variance.

**Figure 9:**
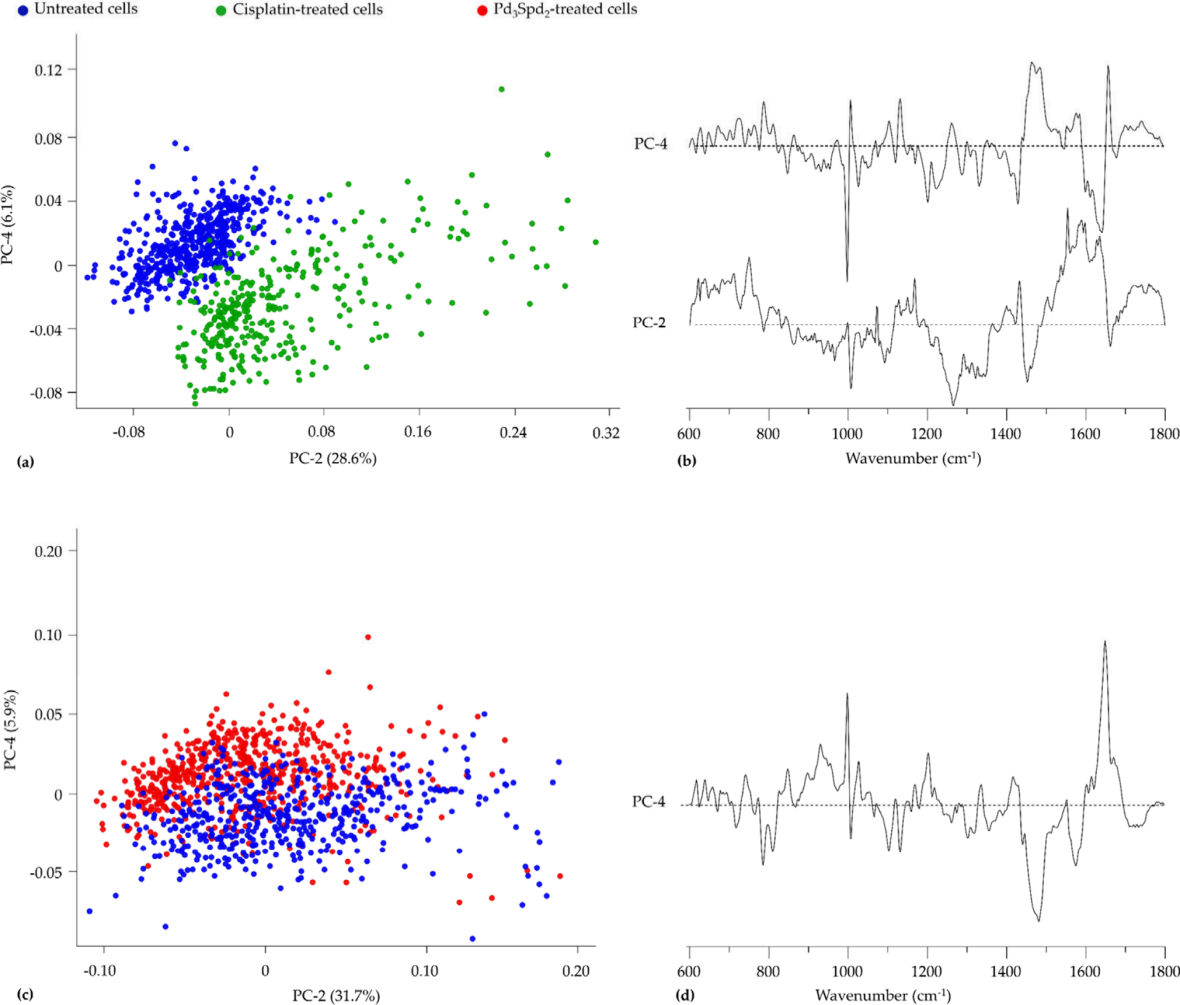
PCA of Raman data for healthy breast cell line MCF-12A treated with (a,b) cisplatin vs untreated cells and (c,d) Pd_3_Spd_2_ vs untreated cells.

Drug-treated cells exhibited a higher discrimination for DNA biomarkers due to the presence of the νCO from RNA/ribose at 1128 cm^–1^, νCC_ring_ from cytosine, guanine, and thymine at 1175 cm^–1^ and the νOPO_backbone_ from A-DNA at 805 cm^–1^ (Table S1). The presence of A-DNA in healthy cells is not in itself a problem, since this conformational change is reversible; however, the change of the νOPO_backbone_ from DNA is an indicator of cell death. By the score and loadings interpretation, the drugs showed a higher impact on the ν_s_CC_ring_ (at 1005 cm^–1^) and δCH, νO–CH_3_ (at 1034 cm^–1^) from phenylalanine, νCN_proteins_ (at 1128 cm^–1^), δCH_2_ from tyrosine and phenylalanine (at 1175 cm^–1^) and νCC from hydroxyproline, phenylalanine and tyrosine (at 1202 cm^–1^). Also, conformational changes in amide I were detected for random coils at 1640–1650 cm^–1^ in the presence of the drugs. On the other hand, untreated cells show a higher contribution from vibrational modes of tryptophan at 677 cm^–1^ (νCC_ring_) and at 723 cm^–1^ (ν_s_CC_ring_). Also, untreated healthy cells have a greater contribution of vibrational modes from nucleic acids at 677 cm^–1^ (B-DNA/A,G,T,C νCC_ring_), at 723 cm^–1^ (B-DNA/A νCC_ring_), at 785 cm^–1^ (B-DNA νOPO_backbone_), and at 1580 cm^–1^ (A,G νCC_ring_).

From the fingerprint region of the FTIR data (Figure 8d,e), it is possible to separate the untreated cells from the treated cells along PC-1 in combination with PC-3, explaining 59.2 and 2.6% of total variance, respectively. The Pd_3_Spd_2_-exposed MCF-12A cells show a higher contribution from amide II (δCN-H/νCN) at 1545 cm^–1^ and amide I/random coil at 1640–1650 cm^–1^. The cisplatin-treated cells and untreated cells presented higher intensity at 1080 cm^–1^ (νCC and νCN from proteins, ν_s_PO_2_^–^ from phospholipids, νCC and νCO from glycogen) at 1407 cm^–1^ (δNH_2_ from proteins), at 1440–1450 cm^–1^ (δCH_2_ from lipids) and at 1580 cm^–1^ (νCC_ring_ from adenine and guanine). According to the results of the FTIR high-wavenumber region (Figure 8g,h), the separation between the three conditions was evident along PC-1, explaining 90.7% of the total data variance. Analyzing the loadings, it was possible to notice changes in the bands assigned to the CH and CH_2_ from lipids (at 2850–2875 cm^–1^), that overlap with CH_2_ from proteins and carbohydrates (Table S1).

According to the dendrograms obtained by HCA (Figure 8), the three conditions can be completely separated into different clusters in both techniques. In FTIR (Figure 8j), untreated cells and cisplatin-treated cells have a higher similarity, which is an indication of the original integrity of the cells. Although the drug has altered some of the biochemical composition of the cell, it still retains characteristics of the cell without the drug. The higher level of similarity between the cisplatin-treated cells and Pd_3_Spd_2_-treated cells was observed in Raman (Figure 8k), which is shown in the PCA through the slight overlaps of both conditions.

The confusion tables from the Raman and FTIR classification model, respectively, relating to the comparison between the MCF-12A cell line untreated and treated with Pd_3_Spd_2_ are depicted in Figure 8c,f,i. The confusion table of the Raman demonstrated that among 541 spectra from the control group, 32 (6%) were misclassified as Pd_3_Spd_2_-treated cells, signifying that 509 spectra were correctly classified as control (Figure 8c). The confusion table of the FTIR fingerprint region indicates that among 479 spectra from the control, only 1 was misclassified as Pd_3_Spd_2_-treated cells (Figure 8f). On the other hand, 478 out 479 spectra were accurately classified as belonging to the Pd_3_Spd_2_-treated cells. Regarding the confusion table of the FTIR high-wavenumber region, 531 out of 550 were correctly classified as the control and only 4% of Pd_3_Spd_2_-treated cells spectra were misclassified as belonging to the control group (Figure 8i).

### Mesenchymal TNBC Cells Treated with Pd_3_Spd_2_

The results gathered for the MDA-MB-231 and MDA-MB-231/R, both treated with Pd_3_Spd_2_, were analyzed and compared (Figure S1, Supporting Information). A clear separation was achieved between these two groups by Raman and FTIR. For the former, discrimination was observed along PC-1 for the fingerprint (44.3%) and also along PC-1 for the high-wavenumber (92.8%) regions, based on the following spectral differences shown in the loading plots (Figure S1b,e): Pd_3_Spd_2_ showed a higher impact on DNA signals at 748 and 930 cm^–1^ (νOPO_backbone_ from the Z-DNA), at 1261 cm^–1^ (νCC_ring_ from RNA/dT), and at 1302 cm^–1^ (νCC_ring_ from RNA/A,C) for cisplatin-sensitive compared to cisplatin-resistant cells (Table S1). Also, proteins at 1005 cm^–1^ (ν_s_CC_ring_ from phenylalanine), at 1080 cm^–1^ (νCC, νCN) and at 1235 cm^–1^ (amide III/β-sheet) presented a higher contribution for cisplatin-sensitive cells. Pd_3_Spd_2_ exhibited a great impact on phospholipids (ν_s_PO_2_^–^) at 1088 cm^–1^ and on δCH_2_ from lipids (at 1433 cm^–1^) for cisplatin-sensitive cells. Therefore, it was clear that the MDA-MB-231/R cells presented a higher contribution from proteins on amide I (at 1640–1670 cm^–1^).

From the FTIR fingerprint data, it is possible to separate the cisplatin-resistant and cisplatin-sensitive cells treated with Pd_3_Spd_2_ along PC-3 (which covers 61.8% of the total variance). The Pd_3_Spd_2_-exposed cisplatin-sensitive cells showed a higher contribution from proteins at 1396 cm^–1^ (δCH_3_, ρCH_2_), at 1613 cm^–1^ (νC=C from phenylalanine, tyrosine and tryptophan) and at 1690 cm^–1^ (νC=O). Regarding the lipid content, Pd_3_Spd_2_ also displayed a higher impact in vibrational modes of membrane lipids (δCH_3_) at 1396 cm^–1^ and (δCH_2_) at 1440–1450 cm^–1^ for cisplatin-sensitive cells. Concerning the FTIR high-wavenumber range, the separation was obtained along PC-1 and PC-3 (78.0% and 1.8% of total variance, respectively). The Pd_3_Spd_2_-induced changes were observed in the typical νCH_2_ (at 2850–2935 cm^–1^) and νCH_3_ (at 2955 cm^–1^) vibrational modes (Table S1).

Figure S1c-l depicts the confusion tables for TNBC cell lines treated with Pd_3_Spd_2_. The confusion table of the Raman fingerprint showed that 82% of spectra were correctly classified as cisplatin-resistant cells treated with Pd_3_Spd_2_ and 92% were classified as cisplatin-sensitive cells treated with Pd_3_Spd_2_ (Figure S1c). Regarding Raman high wavenumbers, only 10% and 8% were misclassified as MDA-MB-231 and MDA-MB-231/R, both treated with Pd_3_Spd_2_, respectively (Figure S1f). Concerning the FTIR fingerprint region, 88% and 86% were accurately classified as belonging to the Pd_3_Spd_2_-treated MDA-MB-231/R and Pd_3_Spd_2_-treated MDA-MB-231, respectively (Figure S1i).

The confusion table of FTIR high wavenumbers indicates that among 635 spectra, 26 were misclassified as Pd_3_Spd_2_-treated MDA-MB-231/R. On the other hand, 69 out 414 spectra were misclassified as Pd_3_Spd_2_-treated MDA-MB-231­(Figure S1l).

## Conclusions

Single-point Raman and FTIR measurements were performed on human healthy breast (MCF-12A) and mesenchymal TNBC (MDA-MB-231 and MDA-MB-231/R) cell lines treated with a Pd­(II) complex with a biogenic polyamine (spermidine), comprising 3 metal centers. Specific vibrational signatures of drug-free and drug-exposed (48 h) cells were obtained. Through multivariate analysis, a clear discrimination between drug-treated and untreated cells was achieved, allowing for reliable spectral biomarkers of drug impact for mesenchymal TNBC to be identified: drug-treated cells evidenced major changes, namely, native B-DNA to A- or Z-DNA conformations. The presence of the Z form of DNA is a biomarker of cell destruction and death since the cell’s repair mechanisms are unable to restore it from Z- to B-DNA. It is possible that polynuclear complexes cause the change from B- to Z-DNA, since they establish several cross-links, not only intrachain but also interchain. These results are in accordance with the generally accepted mode of action for cisplatin-type DNA groove-binding agents. On the other hand, cisplatin showed a more significant impact on proteins. Pd-complex causes a higher impact not only on lipids but also in proteins, namely, in amide I and II. Cisplatin affected healthy cells in the lipidic content, particularly in phospholipid vibrational modes. When comparing cisplatin-sensitive and cisplatin-resistant cells treated with Pd_3_Spd_2_, cisplatin-sensitive cells were the most affected by the drug through most of the cellular components: DNA (B- to Z-DNA conformation), proteins, and lipids. To the cisplatin-resistant cells, a higher contribution of protein bands was found. The results presently gathered are promising regarding the search for new effective and selective chemotherapeutic drugs against mesenchymal TNBC, leading to irreversible damage to cancer cells while maintaining the integrity of healthy cells. In addition, acquired resistance and toxicity effects should be minimized by searching novel pathways of cytotoxicity. MicroRaman and microFTIR are powerful techniques that enable the identification of molecular changes induced by drug exposure, each providing complementary and unique information: FTIR spectra exhibited a higher signal-to-noise ratio than Raman spectra and showed greater sensitivity in detecting changes in lipid and protein content. On the other hand, Raman microspectroscopy provided enhanced structural resolution, particularly for identifying conformational changes in nucleic acids. Furthermore, other techniques could be interesting to complement our results. For instance, cryo-transmission electron microscopy (TEM) could provide high-resolution ultrastructural information on drug-induced morphological changes, while Inductively Coupled Plasma Mass Spectrometry (ICP-MS) would allow quantitative analysis of metal content and drug accumulation at the cellular level. This improved knowledge of the main cellular drug targets constitutes a paramount advance in cancer treatment, particularly regarding a type of malignancy with high morbidity and mortality.

## Supplementary Material



## Notes

Experimental section, table of Raman and infrared bands, and figure of PCA of Raman and FTIR data (PDF)
