# Supplementary material for: New Chemotherapeutic Approaches to Treatment of Mesenchymal Triple-Negative Breast Cancer-Sensitive and Resistant to Cisplatin: Assessment of Cellular Response by Vibrational Microspectroscopy
Source: Anal Chem. 2025 Jul 4;97(27):14709–21. doi: 10.1021/acs.analchem.5c02233 (PMC12818719; doi:10.1021/acs.analchem.5c02233)
Supplement: Supplementary file 1 [file ac5c02233_si_001.pdf]

Supplementary Information

New Chemotherapeutic Approaches to Mesenchymal Triple-Negative Breast Cancer-Sensitive and Resistant to Cisplatin: Assessment of Cellular Response by Vibrational Microspectroscopy

Clara B. Martins[a,b], Ana L. M. Batista de Carvalho[a], Maria M. Félix[a], Martin Vojtek[c], Carmen Diniz[c], Luís A. E. Batista de Carvalho[a], Maria P. M. Marques[a,b]*

[a]Molecular Physical-Chemistry – LAQV/REQUIMTE, Department of Chemistry, University of Coimbra, 3004-535 Coimbra, Portugal; clara.b.martins@uc.pt (C.B.M.); almbc@uc.pt (A.L.M.B.d.C.); mmfelix@uc.pt (M.M.F.) labc@ci.uc.pt (L.A.E.B.d.C.); pmc@ci.uc.pt (M.P.M.M.)

[b]Department of Life Sciences, Faculty of Science and Technology, University of Coimbra, 3000-456 Coimbra, Portugal

[c]LAQV/REQUIMTE, Laboratory of Pharmacology, Department of Drug Sciences, Faculty of Pharmacy, University of Porto, 4050-313 Porto, Portugal; matovoj@gmail.com (M.V); cdiniz@ff.up.pt (C.D.)

[*]Corresponding author

TABLE OF CONTENTS

Experimental Section

Chemicals and Solutions

Synthesis of Pd_3Spd_2

Cell Culture

Sample Preparation for Vibrational Microspectroscopy Measurements

Tables

Figures

References

Experimental Section

Chemicals and Solutions

Antibiotics (penicillin–streptomycin 100x solution), bovine insulin (10 mg/mL insulin in 25mM HEPES, pH 8.2), cholera toxin from *Vibrio cholerae*, cisplatin (cis-dichlorodiammine platinum(II), >99.9%), Dulbecco's Modified Eagle's Medium-high glucose (DMEM-HG, 4500 mgL^{-1} glucose), 1:1 mixture of Dulbecco's Modified Eagle's Medium and Ham's F12 cell growth medium (DMEM/F12 1:1), dimethyl-sulphoxide (DMSO), formalin (10% *v/v* neutral-buffered formalin, *ca.* 4% formaldehyde), human epidermal growth factor (hEGF; recombinant, expressed in *E. coli*), hydrocortisone, phosphate buffered saline (PBS), potassium tetrachloropalladate(II) (K_2PdCl_4, 98%), sodium bicarbonate ($NaHCO_3$, ≥99.0%), spermidine (N^1-(3-Aminopropyl)butane-1,4-diamine, 99%), trypan blue (0.4% *w/v*) and trypsin-EDTA (1x), as well as inorganic salts and acids were purchased from Merck KGaA (Algés, Portugal). Fetal bovine serum (FBS) was obtained from Gibco-Life Technologies (Porto, Portugal). For drug administration, initial stock solutions of Pd_3Spd_2 and cisplatin were prepared in PBS with 10% (*v/v)* DMSO and PBS, respectively. human mesenchymal TNBC MDA-MB-231 cell line (ATCC HTB-26) and the non-cancerous breast cell line MCF-12A (ATCC CRL-10782) were purchased from ATCC (Manassas, VA, USA).

Synthesis of Pd_3Spd_2

The Pd_3Spd_2 complex was synthesised according to a published procedure[1] optimised by the authors: 3 mmol of K_2PdCl_4 were dissolved in a minimal amount of water, and 1.98 mmol of spermidine trihydrochloride aqueous solution was added dropwise, at room temperature, under continuous stirring. After 15 minutes, the resulting orange precipitate was isolated by filtration, washed with water, ethanol and acetone, and air-dried.

The composition and purity of the newly synthesised compounds were verified by elemental analysis and vibrational spectroscopy (FTIR, Raman and inelastic neutron scattering (INS)).

The drug stock solutions of Pd_3Spd_2 and cisplatin were prepared in PBS with 10% (*v/v)* DMSO. All solutions were sterile filtered and stored at -20 °C.

Cell Culture

Resistant mesenchymal TNBC cell line (MDA-MB-231/R) was obtained and cultured as previously described[2], briefly: MDA-MB-231 cells were continuously treated with increasing concentrations of cisplatin (up to 2 μM) during a period of 6 months. When a consistent cell growth rate in the presence of cisplatin was achieved, these cells, hereafter named MDA-MB-231/R, were stocked to ensure the consistency of the phenotype for future experiments. All posterior assays with this cell line were performed within 10 passages, in order to maintain resistance to cisplatin while routinely growing the MDA-MB-231/R cell line in a cell culture medium without the addition of cisplatin[2].

DMEM-HG cell growth medium supplemented with 10% (*v/v*) heat-inactivated FBS and 1.5 g/L sodium bicarbonate (pH 7.4) was used to culture the cisplatin-sensitive breast cancer cells (MDA-MB-231 and MDA-MB-231/R).

MCF-12A cells were cultured in DMEM/F12 medium supplemented with 100 ng/mL cholera toxin, 0.01 mg/mL bovine insulin, 20 ng/mL hEGF, 500 ng/mL hydrocortisone and 5% (*v/v*) horse serum.

All cell lines were cultured in monolayers, at 37 ˚C, in humidified atmosphere of 5% CO_2. Under these conditions, the population doubling time was 20.6 ± 3.1 h, 25.5 ± 0.9 h and 30.6 ± 1.1 h for MCF-12A, MDA-MB-231 and MDA-MB-231/R cells, respectively. The tested agents were added to the cells when these were in the respective exponential phase of growth.

Sample Preparation for Vibrational Microspectroscopy Measurements

Upon harvesting by trypsinization, the cells were centrifuged, and the pellet was resuspended in culture medium and seeded at a concentration of 1.5 x 10^4 cells cm^2 onto optical substrates suitable for either Raman or FTIR acquisition (respectively, MgF_2 (Crystran, 1mm x 13mm) or CaF_2 (Crystran UV-grade, 1mm x 13mm). After an incubation period of 24 hours (allowing the cells to attach to the substrate), the cells were treated with either Pd_3Spd_2 or cisplatin at the respective IC_{50} values (50% cell growth inhibition value) - previously determined by the authors[2], respectively for Pd_3Spd_2 and cisplatin – 4.65 μM and 1 μM for MDA-MB-231, 10.57 μM and 32.4 μM for MDA-MB-231/R, 53 μM and 1 μM for MCF-12A. Upon 48h of drug exposure, the culture medium was removed, the cells were washed with PBS and fixed for 10 min with 4% formalin (diluted in 0.9% NaCl from the commercial neutral buffered formaldehyde solution). After repeated washing with Mili-Q water, the disks were air-dried prior to spectroscopic analysis. All samples were prepared in triplicate, in three independent assays.

The cells were chemically fixed with 4% formaldehyde solution (formalin). Although this chemical fixative causes crosslinking between the aldehyde and the primary and secondary amine groups of cellular proteins, it maintains the cell integrity as similar as possible to *in vivo* conditions, for prolonged time periods, avoiding contaminations in the fixation process. Some effects of fixation are expected, such as some reduction in the signal intensity, due to the conformation changes of proteins and lipid assembly disruption[3, 4]. However, the vibrational modes of formalin were not found in the spectral profile (a t 907, 1040 and 1490 cm^{-1}), showing that fixation had a weak impact on the overall molecular content[5-7].

Tables

Table S1. Raman and infrared bands for human breast healthy and cancer cells: MCF-12A, MDA-MB-231 and MDA-MB-231/R, respectively. (The drug-affected bands are represented in bold. The signals exclusively detected by infrared are shaded in grey).

Band (cm^{-1})	Assignment[a]			
	**Nucleic Acids**	**Proteins**	**Lipids**	**Carbohydrates**
621		Phe (τCC)		
645		νCS, Tyr (τCC)		
**669**	**A-DNA/dG (νCC_{ring})**			
677	B-DNA/A,G,T,C (νCC_{ring})	Trp (νCC_{ring})		
697-900			Phosphate esters (ν_sOPO)	
698	B-DNA/dG (νCC_{ring})	Met (νCS)		
723	B-DNA/A (νCC_{ring})	Trp ($\nu_s CC_{ring}$)		
749	B-DNA/T (νCC_{ring})			
760	B-DNA/dT (νCC_{ring})	Trp ($\nu_s CC_{ring}$)		
777	C,T,U (νCC_{ring})			
785	B-DNA ($\nu OPO_{backbone}$)			
**808**	**A-DNA ($\nu OPO_{backbone}$)**			
830	B-DNA ($\nu OPO_{backbone}$)	Pro, Tyr (νCC)		
**835-840**	**Z-DNA ($\nu OPO_{backbone}$)**			Glu (δCCO)
855		Pro, Tyr, Val (νCC), δCCH)		polysaccharides (γCOC)
863		Pro (νCC)		mono, disaccharides (νCOC)
880	RNA (ribose, (νCC_{ring})	Tyr (νCC_{ring}), Hyp (νCC)	Phosphocoline ($\nu_s CCN^+$)	νCC, $\nu C\text{-}O_{ring}$
881		νCC	νCC	
903	deoxyribose (νCC_{ring})	νCC	Fatty acids (νCC, νCO)	νCOC
920-930	B-DNA/deoxyribose (νCC_{ring})			
940	RNA/ribose (νCC_{ring})			polysaccharides (skeletal modes)
1005		Phe ($\nu_s CC_{ring}$)		
1034		Phe (δCH, $\nu O\text{-}CH_3$)	νCC, phospholipids (δCH)	νCC, νCO, νC-OH
1063	B-DNA/deoxyribose (νCO)	νCC, νCN	νCC, νCO	νCC, νCO, δOCH
1080		νCC, νCN	phospholipids ($\nu_s PO_2^-$)	glycogen (νCC, νCO)
1092	B-DNA ($\nu_s PO_2^-$)			
1095			νCC, νCN	νCC, νCO
1099	A-DNA ($\nu_s PO_2^-$)			
1128	RNA/ribose (νCO)	νCN	νCC_{acyl} (*trans* conformation)	νCO, νCC
1158		νCC, νCN, δCH_2	δCH_2, $=C\text{-}C=_{conjugated}$	δCH_2
1175	C,G,T (νCC_{ring})	Tyr, Phe (δCH)		
1202		Hyp, Phe, Tyr (νCC)		
1238	B-DNA ($\nu_{as} PO_2^-$)			
1242-1264	A,T (νCC_{ring})	δCH_2, δC=C-H	δCH_2, $\delta C{=}C\text{-}H_{phospholipids}$	δCH_2, ωCH_2, tCH_2
1244-1258	C (δNH_2)			
1252-1264	RNA/dT (νCC_{ring})			
1258	dC (νCC_{ring})			
1270	RNA/C,U (νCC_{ring})	ωCH_2	phospholipids (δC=C)	ωCH_2
1272		Amide III/α-helix	ωCH_2, tCH_2	ωCH_2, tCH_2
1302	RNA/A,C (νCC_{ring})			
1313	G (νCC_{ring})	ωCH_2, tCH_2	ωCH_2, tCH_2	ωCH_2, tCH_2
1338	G (νCC_{ring})			

1373	A,G,T (νCC_{ring})	glycoproteins (δCH_3)	lipids/acyl chains (δCH_3)	saccharides (δCH_2)
1396		$\delta_s CH_3$	membrane lipids ($\delta_s CH_3$)	
1400		$\delta_s CH_3$	$\delta_s CH_3$	
1407		δNH_2		
1420-1480		δCH_2, δCH_3	δCH_2, δCH_3, aromatic lipids	δCH_2
**1420**	**A-DNA (δCH_2)**			
**1425**	**Z-DNA (δCH_2)**			
1440-1450			δCH_2	
1516	C (νCC_{ring})			
1545		Amide II (δCN-H/ νCN)		
1580	A,G (νCC_{ring})			
1608-1619	A (νCC_{ring}), C (δNH_2)	Phe, Tyr, Trp (νC=C), $\delta(NH_2)$		
**1612-1690**		**Amide I/β-sheet, Antiparallel**		
**1626-1640**		**Amide I/β-sheet, Parallel**		
**1640-1650**		**Amide I/random coil**		
1650-1660	DNA (δNH)	Amide I (νC=O)/ α-helix	νC=C	
1690		$\nu C{=}O_{amino\ acid\ side\ chain}$		
**1714**	**A-DNA (νC=O)**			
1718	B-DNA (νC=O)			
1724-1732			phospholipids ($\nu C{=}O_{ester}$)	
2850-2875		$\nu_s CH_2$	$\nu_s CH$, $\nu_s CH_2$	$\nu_s CH$, $\nu_s CH_2$
2880		$\nu_s CH_3$	$\nu_s CH_3$	$\nu_s CH_3$
2900-2935		$\nu_{as} CH_2$	$\nu_{as} CH_2$	$\nu_{as} CH_2$
2955		$\nu_{as} CH_3$	$\nu_{as} CH_3$	$\nu_{as} CH_3$
3069		Amide B		
3250-3295		Amide A (νNH)		
3300-3350			νOH	νOH

[a]A – adenine; C – cytosine; dG – deoxyguanine; dT – deoxythymine; G – guanine; Glu – glucose; Hyp – hydroxyproline; Met – methionine; Phe – phenylalanine; Pro – proline; T – thymine; Trp – tryptophan; Tyr – tyrosine; U – uracil; Val – valine. δ – in-plane deformation; γ – out-of-plane deformation; ν – stretching; ρ – rocking; t – twisting; ω – wagging. s – symmetric; as – anti-symmetric.

Figures

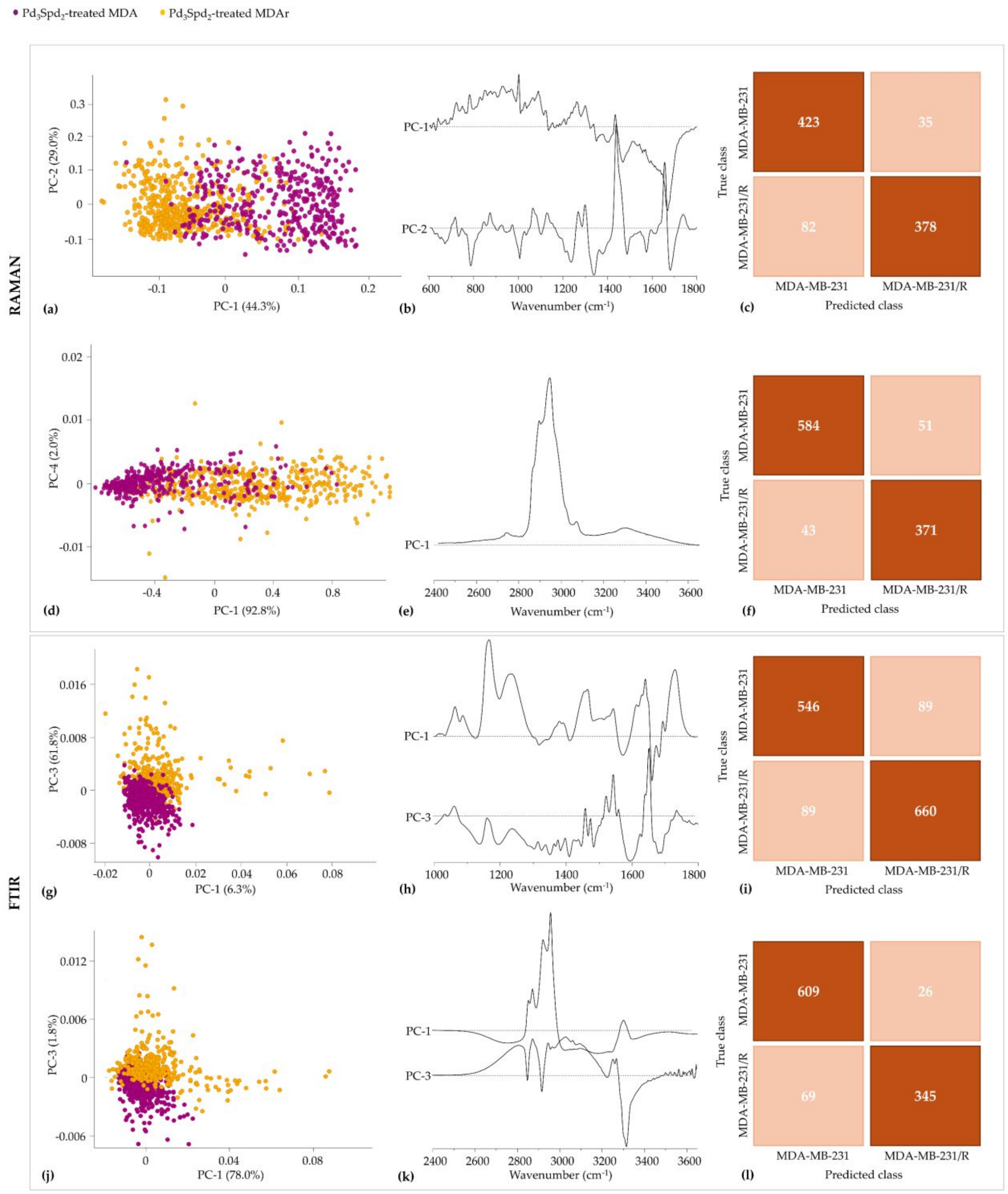

Figure S1. PCA of Raman and FTIR data for TNBC cell lines treated with Pd_3Spd_2. (**a**,**b**) Score and loading plots of the Raman fingerprint region. **(d,e)** Score and loading plots of the Raman high wavenumber region. **(c,f)** Confusion tables of the Raman fingerprint **(c)** and high wavenumber **(f)** classification model tested on Pd_3Spd_2 data. (**g**,**h**) Score and loading plots of the FTIR fingerprint region. (**j**,**k**) Score and loading plots of the FTIR high wavenumber region. (**i**,**l**) Confusion tables of the FTIR fingerprint **(i)** and high wavenumber **(l)** classification model tested on Pd_3Spd_2 data.

References

1. F. Z. C. Navarro-Ranninger, J. M. Perez, I . Ldpez-Solera, S. Martinez-Camera, J . R. Masaguer, and C . Alonso, *Journal of Inorganic Biochemistry*, 1992, **46**, 267-279.
2. M. Vojtek, C. B. Martins, R. Ramos, S. G. Duarte, I. Ferreira, A. L. M. Batista de Carvalho, M. P. M. Marques and C. Diniz, *Pharmaceutics*, 2023, **15**.
3. E. Gazi, J. Dwyer, N. P. Lockyer, J. Miyan, P. Gardner, C. Hart, M. Brown and N. W. Clarke, *Biopolymers*, 2005, **77**, 18-30.
4. M. J. Baker, J. Trevisan, P. Bassan, R. Bhargava, H. J. Butler, K. M. Dorling, P. R. Fielden, S. W. Fogarty, N. J. Fullwood, K. A. Heys, C. Hughes, P. Lasch, P. L. Martin-Hirsch, B. Obinaju, G. D. Sockalingum, J. Sule-Suso, R. J. Strong, M. J. Walsh, B. R. Wood, P. Gardner and F. L. Martin, *Nat Protoc*, 2014, **9**, 1771-1791.
5. J. W. Chan, D. S. Taylor and D. L. Thompson, *Biopolymers*, 2009, **91**, 132-139.
6. F. Draux, C. Gobinet, J. Sule-Suso, A. Trussardi, M. Manfait, P. Jeannesson and G. D. Sockalingum, *Anal Bioanal Chem*, 2010, **397**, 2727-2737.
7. A. J. Hobro and N. I. Smith, *Vibrational Spectroscopy*, 2017, **91**, 31-45.
